# Structural lineament analysis of the Bir El-Qash area, Central Eastern Desert, Egypt, using integrated remote sensing and aeromagnetic data

**DOI:** 10.1038/s41598-023-48660-x

**Published:** 2023-12-07

**Authors:** Waheed H. Mohamed, Mahmoud H. Elyaseer, Mohamed Elsadek M. Sabra

**Affiliations:** 1 Geology Department, Faculty of Science, Al-Azhar University, Cairo, Egypt; 2 Egyptian Mineral Resources Authority, PO. Box 11517, Abbassiya, Cairo Egypt

**Keywords:** Environmental sciences, Solid Earth sciences

## Abstract

The Bir El-Qash area, located in the Central Eastern Desert of Egypt, is characterized by a diverse range of igneous, metamorphic, and sedimentary rocks with ages spanning from the Late Proterozoic to Quaternary. Integration of remote sensing with aeromagnetic data was conducted to generate surface and subsurface structural lineaments. Shaded relief from digital elevation models, principal component analysis of Landsat-8 data, and ALOS/PALSAR images were utilized to create lineament maps. Airborne magnetic data were employed to reveal subsurface characterizations. The study area has undergone various tectonic activities, resulting in complex structures. Multiple fault trends and fractures were identified, including the NW–SE (Red Sea-Gulf of Suez) trend, the NE–SW trending Syrian arc trend, the N–S trending East African trend and the WNW–ESE trend. By analyzing the tectonic features of the Bir El-Qash area, this study provides insights into the geological history and evolution of the Eastern Desert of Egypt.

## Introduction

Remote sensing and geophysical data integration have been integral to accurately mapping lineaments. By utilizing remote sensing technologies such as digital elevation models and Landsat-8 data, alongside geophysical data such as airborne magnetic data, researchers can gain a comprehensive understanding of both surface and subsurface tectonic features. This integrated approach has proven to be a powerful tool in geological exploration and analysis^1^.

Lineament mapping is an important aspect of geological studies as it helps in identifying and understanding the structural elements of the subsurface. In recent years, remote sensing technologies have emerged as a valuable tool for lineament mapping. In particular, shaded relief maps were developed from the digital elevation models (DEMs) used for identifying the lineaments. DEMs enhanced the lineaments by using eight hill shade maps with different sun angles. Combining the first group of hill shade maps (East maps) into the final map also combines the second group of hill shade maps (West maps) into the final map. Landsat-8 data and ALOS/PALSAR images have been utilized, and the principal component analysis technique has been applied to both data to create lineament maps.

The main objective of an aeromagnetic survey is to identify the subsurface geological setting based on variations in the earth's magnetic field that appear as anomalies as a result of the contrast in the magnetic susceptibility between the basement complex and the sedimentary cap^2^. The magnetic method is used in different applications, such as locating faults, folds, shear zones, contact, and determining geological structures that may play a role in mineral exploration. Magnetic maps of the area are then used to identify tectonic settings and structures within the sediments through various advanced interpretation methods like filtering, power spectrum analysis, tilt derivatives, Euler deconvolution, and source parameter imaging^3,4^.

**Figure 1:**
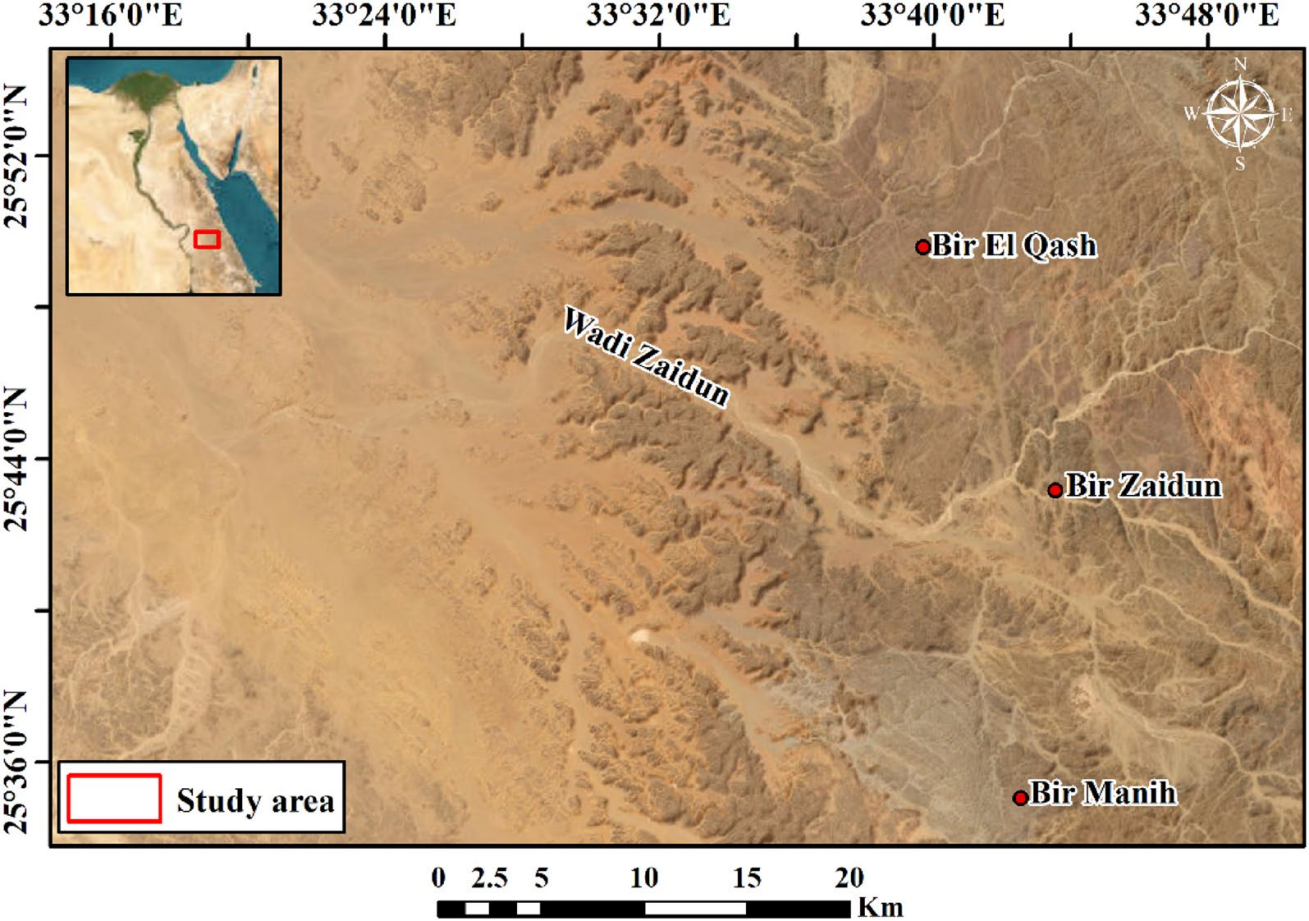
Location map of the Bir El-Qash area, Central Eastern Desert, Egypt (using Arc GIS 10.8 and ENVI 5.3 software).

The area under investigation is located in the central part of the Eastern Desert and lies between longitudes 33° 14′ 19.47″ E and 33° 50′ 37.65″ E and latitudes 25° 33′ 40″ N and 25° 54′ 41.11″ N (Fig. 1). The study area has many natural resources that allow sustainable development plans; this makes the area suitable for comparative analysis of available data. The current study aims to exploit remote sensing and airborne magnetic data to determine the structural lineaments that may have a role in mineral exploration in the study area.

## Geologic setting

**Figure 2:**
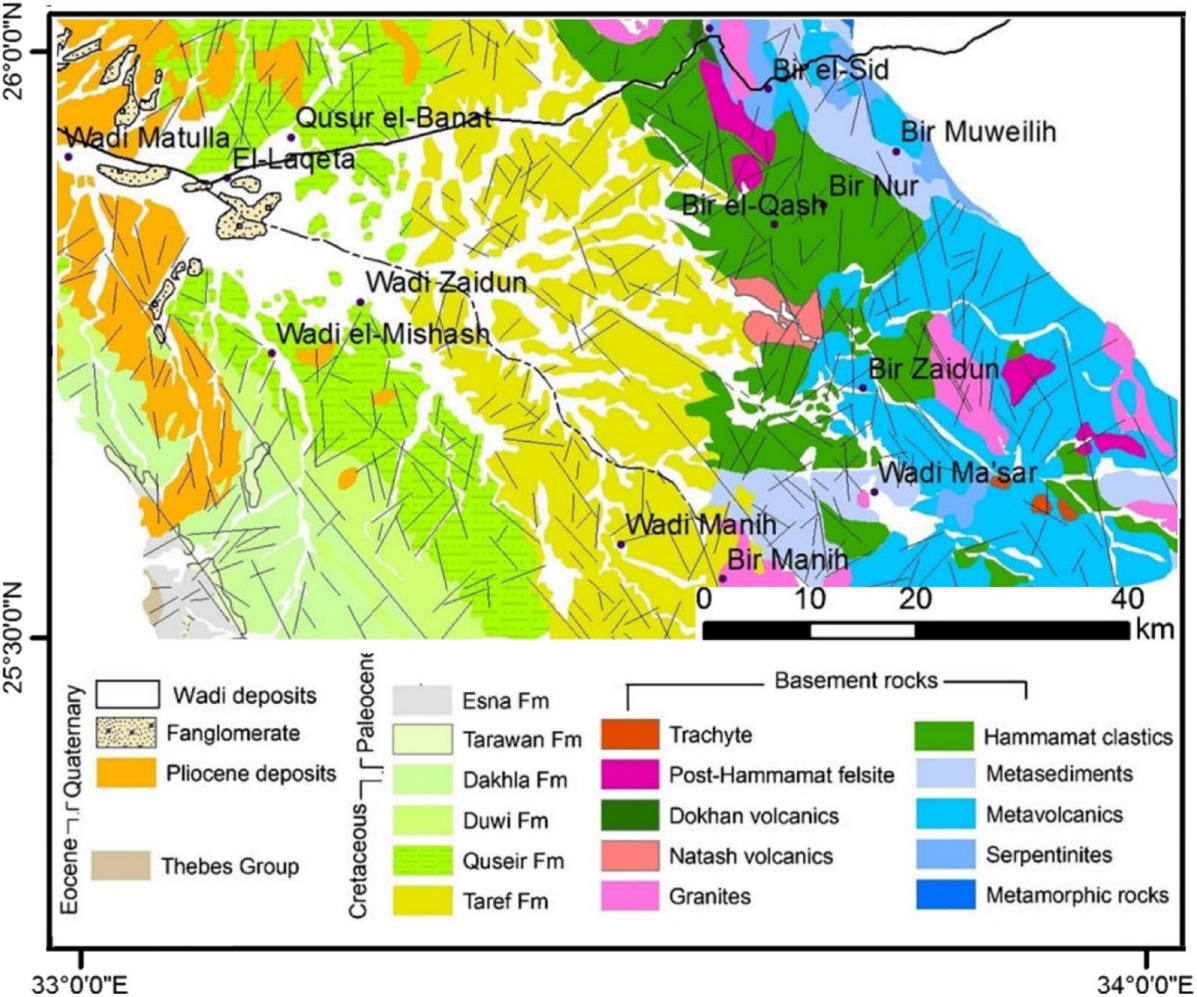
Geological map of the Bir El-Qash Area, Central Eastern Desert, Egypt after^7,23^ (using Arc GIS 10.4).

**Figure 3:**
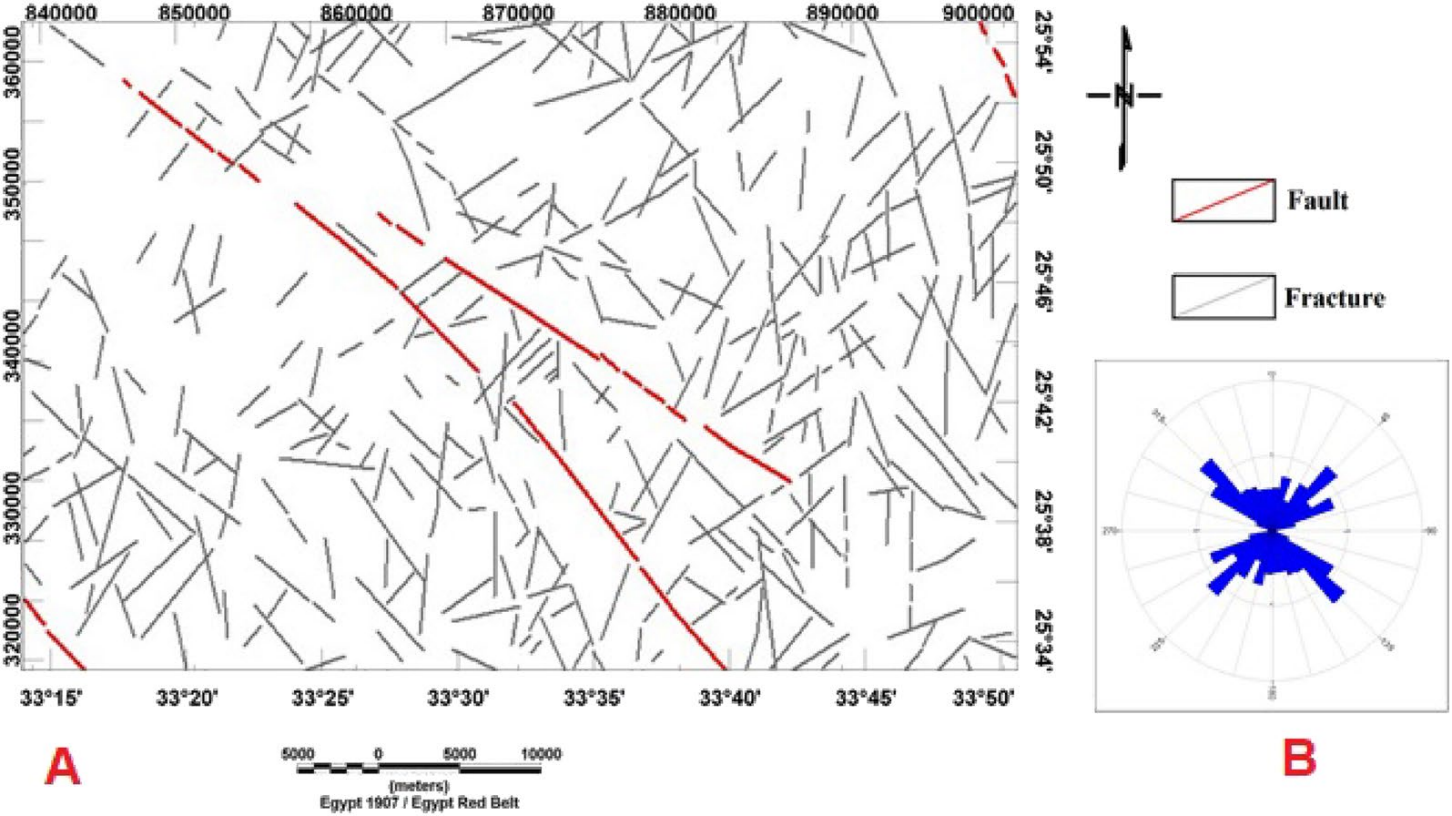
(**A**) Geologic fault trends and fractures derived from the geologic map of Bir El-Qash area, Central Eastern Desert, Egypt^7^ and (**B**) Rose diagram of geologic lineaments of the study area.

The area was intensively investigated by many workers:^5–16^. The regional rock units and the observed structures investigated in the study area are shown **(**Figs. 2, 3**). **The lithostratigraphy of the study area is divided into:

A) Late Proterozoic rocks include the (i) Ophiolite group, which is made up of serpentine talc carbonate and other related rocks; Undifferentiated metavolcanics that are intermediate to acidic; Shelf sediments that have been changed through metamorphosis; and Pyroclastics. The ophiolitic rocks appear as a long belt stretching NW–SE with generally moderate to high relief ^14^; (ii) Hammamat Clastics are essentially unmetamorphosed conglomerates, greywackes, sandstone, and siltstone. The basins include deformed molasse sequences (Hammamat Group) with thicknesses of several thousand meters. More than twenty-five Hammamat basin sites have been discovered in Egypt’s Eastern Desert. The (CED) basins of Wadi Hammamat, Zeidun, El Qash, Arak, Zeidun, Um Esh-Um Seleimat, Queih, El Mayah, Kareim, and Igla are the largest and most well-studied examples^11,12,17–20^; (iii) Younger granitic rocks: are represented by weakly deformed calk-alkaline granitic rocks that are composed of pink coarse-grained biotite perthite syenogranite, and overlain by pink grey, coarse- to medium-grained equigranular biotite monzogranite and alkaline generally undeformed granitic to alkali-feldspar granitic rocks; (iv) Natash volcanics, which are composed of undeformed volcanic rocks that range from basic to acidic alkaline in composition; (v) Dokhan volcanics; these rocks mostly unmetamorphosed that are well known as purple colored rocks (These rocks are named as imperial porphyry and used as antique in the Romanian age). These rocks are represented by rhyodacite, and plagioclase-phyric andesite subvolcanics^21^; (vi) Post-Hammamat rocks, which are primarily represented by felsite and quartz veins that overlain by a series of trachyte plugs and sheets.

B) The Cretaceous rocks in the examined area are subdivided into three units, arranged geochronologically as following from the oldest to the youngest: (i) Taref Formation: is composed of red, brown, medium- to coarse-grained ferruginous cross-bedded sandstone and pebble beds; (ii) Quseir Formation: is made up of coastal varicolored shale, siltstone, and flaggy sandstone, with mixed marine and freshwater fossils of gastropods, pelecypods, plants, and vertebrates; (iii) Dawi Formation: is built up of three phosphate horizons separated by beds of marls, shale, and Oyster limestone with flint. The lower horizon of phosphate is known as the Hamadat or Abu Shigeila horizon, which is followed by a section of variegated shales of non-marine to marginal marine origin. The middle horizon is the best-developed and most consistent of the phosphate horizons that are exploited in the Duwi and Hamadat mines. The phosphatic rock is dark in color and has silicified phosphatic nodules. The upper phosphate horizon named Atshan horizon is explored in the Atshan and an-Nakhil mines in the Quseir area; (iv) Lower part of Dakhla Formation is composed of dark gray shallow marine marl and shale with limestone intercalations.

C) Cenozoic rocks are composed of the following units, from bottom to top: (i) Upper part of the Dakhla Formation; (ii) Tarawan Chalk Formation; this Formation is made up of marl and marly limestones bearing Gryphaea vesicularis; (iii) Esna Formation; is made up of grey laminated shales of about 47–50 m thick in Gabal Duwi and Safaga consequently. The formation ranges in age from the late Paleocene to early Eocene times; (iv) Thebes Formation, which consists of fossiliferous limestone (Oyester beds) with flint and chert concretions interbed. The Thebes Formation reveals the following three-stage depositional history^22^: First stage: basin-wide pelagic carbonate deposition with thin, shallow-water facies near the basin margin; second stage: gradual shallowing and establishment of widespread benthonic communities during intermittent pelagic deposition; simultaneous with tectonic uplift of block faults in the east, which produced many small carbonate platforms separated by deeper basins and third stage: abrupt lowering of sea level, the establishment of shallow-water organic buildups within the basin, and exposure and erosion of uplifted fault blocks to the east; v) Pliocene deposits (Issawiya Formation): consist of fluviatile siltstone, sandstone, and claystone; vi) Quaternary deposits, raised beaches, alluvial fans, and sand covered by wadi deposits.

Structurally, the investigated area was subjected to different tectonic movements, giving rise to some complex structures^12^; (i) The area was developed through three compression deformation phases, followed by a fourth phase of extension and wrenching. D1: includes SW-ward and their accompanied NW–SE imbricate, D2: is represented by N–S, D3: is manifested by NE–SW trending, and D4 is of transtensional nature and is represented by several episodes of normal and strike-slip faulting; (ii) The normal faults have three principle trends, NE–SW, NW–SE, and N–S, which controlled the intrusion of post-Hammamat felsites, post-granite dykes, and post-tectonic granites, respectively; (iii) the strike-slip faults include NNW-dextral and NNE-sinistral faults dissecting every previously developed structure.

## Materials and methods

### Remote sensing data and methods

The main objective of this study is to extract surface geologic structures within the study area. A range of remote sensing data and techniques were employed to evaluate the most efficient method for achieving this goal. Specifically, Digital Elevation Models (DEM), Landsat-8, and ALOS/PALSAR data were utilized.

#### DEM

The SRTM project has produced digital elevation models (DEMs) across a large portion of the globe, from 56° south to 60° north. This has resulted in the creation of the most detailed and comprehensive high-resolution topographic database available on Earth^24^. The SRTM DEM data can be downloaded from http://earthexplorer.usgs.gov.

**Figure 4:**
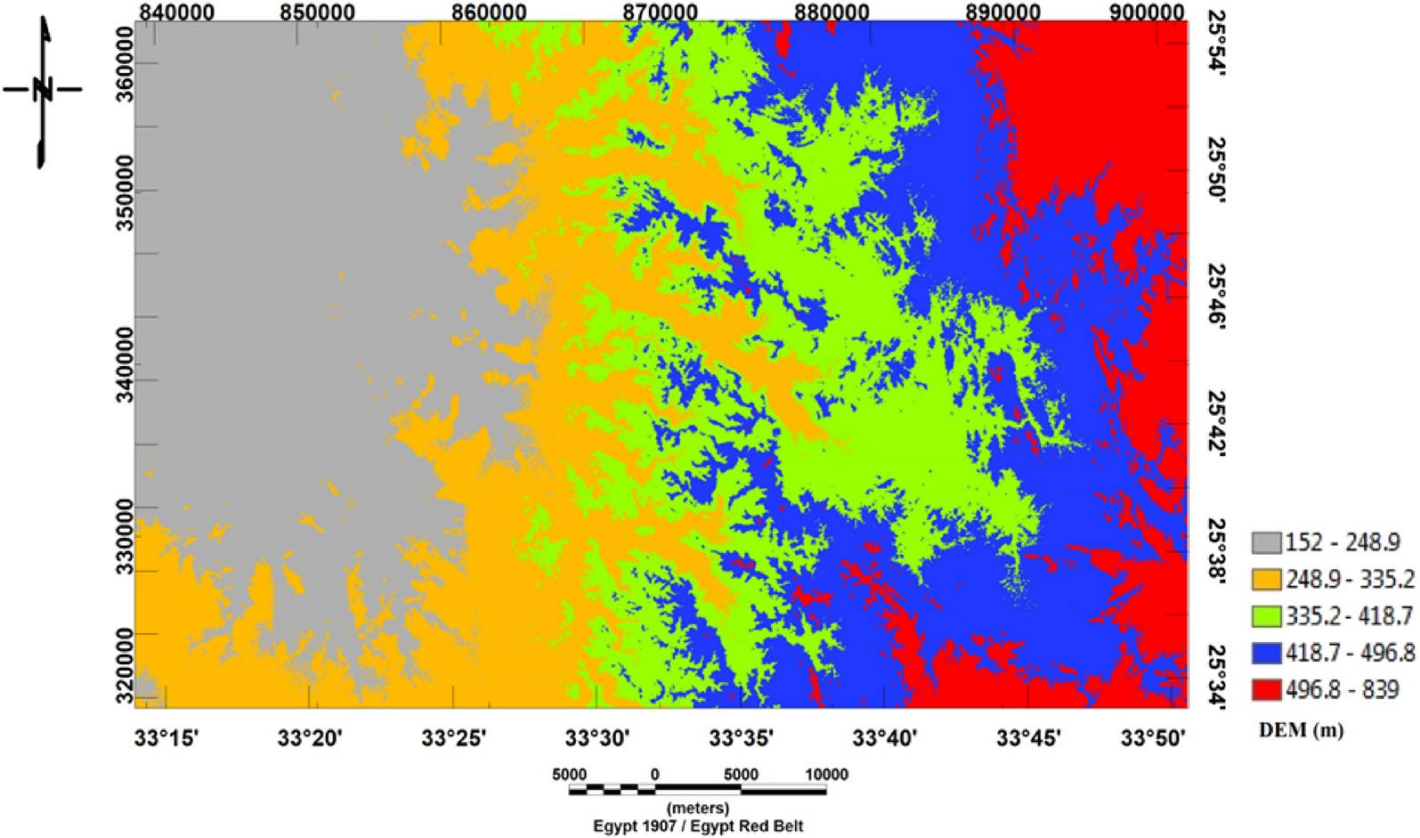
Digital Elevation Model (DEM) of Bir El-Qash area, Central Eastern Desert, Egypt.

To distinguish topographic features from the DEM (Fig. 4), the research area's maximum elevation is found in the northeastern, eastern, and southwestern regions, while the lowest altitude is found in the western parts.

#### Landsat-8 data

This study utilizes data from the OLI and TIRS instruments on the Landsat-8 sensor. Only seven bands of the OLI data were employed, which measure reflected radiation in the VNIR and SWIR regions (bands 2–7) with a spatial resolution of 30 m and the panchromatic band 8 with a spatial resolution of 15 m (Table 1). The deep blue visible channel (band 1) and shortwave infrared channel (band 9) were not utilized as they are primarily designed for water resources, coastal zone investigation, and detecting cirrus clouds, respectively. The thermal bands of the Landsat-8 (TIR), which collect emitted radiation in two bands (10 and 11), were not used in this study. Two cloud-free images were obtained for the same date from the US Geological Survey website (http://earthexplorer.usgs.gov) and are Level 1 Terrain-corrected (L1T) products. The images were geometrically corrected, and a mosaic of VNIR-SWIR data was constructed and preprocessed using ENVI 5.1 software.

**Table 1:** The radiometric characteristics of both optical and microwave data.

Sensor	Bands	Spectral region	Wavelength (µm)	Resolution (m)	Swath width (km)
Landsat-8 OLI/TIRS	Band 1	Coastal	0.433–0.451	30	185
	Band 2	Blue	0.452–0.512		
	Band 3	Green	0.533–0.590		
	Band 4	Red	0.636–0.673		
	Band 5	NIR	0.851–0.879		
	Band 6	SWIR	1.566–1.651		
	Band 7		2.107–2.294		
	Band 8	Panchromatic	0.503–0.676	15	185
	Band 9	Cirrus	1.363–1.384	30	
	Band 10	TIR-1 TIR-2	10.60–11.19	100	
	Band 11		11.50–12.51		
ALOSE/PALSAR	L-Band	Fine Beam Single (FBS) HH	15–30	10	70

Pre-processing is an essential step in this study as it minimizes sensor, solar, atmospheric, topographic effects and distortion for surface reflectance analysis^25^. The FLAASH algorithm was utilized to correct the atmospheric effects and convert radiance data to reflectance in the Landsat-8 OLI data. The process was carried out by utilizing the algorithm on radiance data that had been radiometrically calibrated in the format of band interleaved by line (BIL)^26^.

#### ALOS/PALSAR data

In this research, the Phased Array Type L-Band Synthetic Aperture Radar (PALSAR) Fine Beam Single (FBS) Polarization Mode (HH) was also employed due to its unique radar wavelength (23.62 cm with 1.27 GHz) (Table 1). SAR images are more effective than optical images in identifying subsurface features as they have a longer wavelength, which allows for subsurface penetration^27,28^. The ability of waves to penetrate a surface layer is related to the amount of scattering loss present in the layer. Factors that affect this scattering loss include the grain size being smaller than 10% of the wavelength of the incident wave and the moisture content being less than 1%. The skin depth of the radar L-Band was calculated at ~ 1.5–2 m in non-vegetated dry sand areas, which allows for the detection of buried (invisible) structural features.

In this study, Digital Elevation Models (DEMs), the principal component band (PC1) of Landsat-8 imagery, and the PALSAR band were utilized to extract lineaments. To identify linear topographical features within the Digital Elevation Model (DEM), eight shaded relief images were generated by illuminating the image from eight distinct directions.

#### Principal component analysis (PCA)

The primary technique commonly employed for remote sensing data involving multivariate statistics and dimensionality reduction is known as principal component analysis (PCA). PCA is utilized to generate uncorrelated bands, separating noise elements and reducing the spectral complexity of the data. In order to get the lines of the study area, principal component analysis (PCA) was used to change the VNIR and SWIR bands of Landsat-8 (OLI).

### Magnetic data

The magnetic measurements were carried out in 1982 and recorded in Sheet No. 65. The collaboration between the Egyptian General Petroleum Corporation, the Egyptian Geological Survey and Mining Authority, and the Aero-Service Division of the Western Geophysical Company of America provides aeromagnetic data in the research region. This partnership carried out an airborne magnetic survey over a large portion of Egypt's Eastern Desert, as well as certain portions of the central Western Desert, in 1982 to provide data to help in the identification and assessment of the region's minerals, petroleum, and groundwater resources^29^. The traverse lines were spaced 1.5 km apart and ran in the N45°E direction. The tie lines were roughly 10 km apart and perpendicular to the traverse lines. The separation between stations was 92.65 m, and the aircraft's average speed ranged from 222 to 314 km per hour, with a mean terrain clearance of 120 m. The aeromagnetic data were subjected to different processing techniques, including reduction to magnetic pole (RTP), 2D average power spectrum, regional and residual separation, vertical and tilt derivative, 3D Euler deconvolution, Enhanced horizontal gradient amplitude, and interpreted structural lineaments.

#### Reduction to magnetic pole (RTP)

The RTP technique is a valuable method used to eliminate the influence of magnetic inclination on magnetic data. By transforming inclined magnetic data to a hypothetical vertical field, RTP eliminated anomaly asymmetry caused by inclination and enabled the precise localization of anomalies immediately above their causative sources. According to^30^, the field reduced to the pole at a fixed point above the measurement plane in the frequency domain is given by:

1 $$ {\text{L}}\left( \theta \right) = {1}/\left[ {{\text{sin }}\left( {{\text{I}}_{\alpha } } \right) - {\text{i cos }}\left( {\text{I}} \right){\text{ cos }}\left( {{\text{D}} + \, \theta } \right)} \right]^{{2}} ,\;{\text{if }}\left( {{\text{I}}_{\alpha } < {\text{ I}}} \right),\;{\text{I}}\alpha = {\text{I}} $$

where, I = Geomagnetic inclination, I_*ɑ*_ = Inclination for amplitude correction (never less than I), and D = geomagnetic declination, *θ* = wavenumber direction, i = imaginary component.

Reduction to the pole has an amplitude component (the sin term) and a phase component [the icos (I).cos (D + θ) component]. An amplitude inclination of 90º causes only the phase component to be applied to the data (no amplitude correction), and a value of 0º (zero) causes phase and amplitude components to be applied over the entire range^31^.

#### Radially averaged power spectrum

The application of spectral analysis to gravity and magnetic data has been widely utilized during the last few years to determine the depth of specific geological characteristics, such as the magnetic basement^32^. The power spectrum of magnetic data is just the product of the two-dimensional Fourier transform of the map and its complex conjugation. It is a function of wavelength in both the x and y directions. While traditional depth estimation methods examine individual sources, spectral analysis techniques can provide depth information on networks of anomalies, such as collections of bodies. Since the depth of a single anomaly is less significant, it might not produce results as precise as an average depth. The spectrum analysis method is typically used because it is more accurate than other depth computation methods at determining the average depth of the basement. To calculate these depths, one can utilize the slope of a line that has been fitted to any linear section of the curve using equation^33^.

2 $$ h \, = - S \, / \, 4 \, \pi $$

where, h = depth S = slope of the log (energy) spectrum. These estimates can be used as a rough guide to the depth of the gravity or the magnetic source.

#### Regional and residual separation

Filtering magnetic data is a necessary step before analyzing and interpreting the RTP map. The main objective of the filtering procedures is to prepare the data set and present it in a way that makes it simpler to understand the significance of magnetic anomalies in relation to their geological origins^34^. A method called regional-residual separation can be applied to observed magnetic data to separate it into two parts: shallow sources that are local in nature and regional sources. This technique allows for a better understanding of the magnetic data by separating it into distinct components. In the present study, the Fast Fourier Transform (FFT) was applied to the total magnetic intensity data, using the Oasis Montaj package^31^ to explore the frequency content of these data. The process of regional-residual separation was conducted by applying a Gaussian filter with a cutoff wavelength of 15 km in order to extract the magnetic signature. The Gaussian filter possesses the capability to do either low-pass or high-pass filtering, based on the specific parameters employed during the calculations.

#### First vertical derivative

The vertical derivative (vertical gradient) is a good method for resolving anomalies over individual structures in total magnetic intensity data and, more importantly, suppressing the regional content of the data^35^. It also makes anomalies smaller in width and matches the causative body more closely. Vertical derivative transforms are intended to facilitate the interpretation of reduced-to-pole magnetic data. They are enhancement techniques that amplify the shorter wavelength anomalies relative to those with longer wavelengths. The nth-order vertical derivatives for a given potential field (x, y, z) are calculated as follows:

3 $$ \frac{{\partial {\text{nf}}\left( {{\text{x}},{\text{y}},{\text{z}}} \right)}}{{\partial {\text{zn}}}} = F^{ - 1} [{\text{ K}}\left| {\text{n}} \right|{\text{ F}}\left( {{\text{f}}\left( {{\text{x}},{\text{y}},{\text{z}}} \right)} \right] $$

where F refers to the Fourier transform operator, F^−1^ refers to the inverse Fourier transform operator, and

4 $$ {\text{K}} = \sqrt {{\text{K}}^{2} {\text{x}} + {\text{K}}^{2} {\text{y}}} $$

where k_x_ and k_y_ are the wavenumbers in the x and y directions, respectively. Clearly, multiplying the transformed potential by k to any power will magnify short-wavelength features of the potential field typically associated with near-surface sources while attenuating long-wavelength components.

#### Tilt derivative (TDR

The tilt derivative method (TDR) is employed to enhance the edges of magnetic sources. The TDR filter was suggested and developed by^35–37^. The vertical derivative is divided by the total horizontal derivative to calculate this filter^38^ as follows:

5 $$ TDR = {\text{ tan}}^{{ - {1}}} (VDR/THDR) $$

The tilt angle responses can range from positive values over the source to zero at or near the edge to negative values outside the body. When attempting to determine the relative contrast in magnetization, this sign variation is particularly important because of its ability to provide more details^39^. The TDR approach brings out characteristics that are near the surface in magnetic fields.

#### (3-D) Euler deconvolution

Euler deconvolution is a data enhancement technique for estimating the location and depth of a magnetic anomaly source. It relates the magnetic field and its gradient components to the location of the anomaly source with the degree of homogeneity expressed as a structural index, and it is a suitable method for delineating anomalies caused by isolated and multiple sources^40^. Euler deconvolution is expressed in Eq. 6.

6 $$ \left( {X - X_{0} } \right)\frac{\partial T}{{\partial X}} + \left( {y - y_{0} } \right)\frac{\partial T}{{\partial y}} + \left( {Z - Z_{0} } \right)\frac{\partial T}{{\partial Z}} = n\left( {B - T} \right) $$

Applying Euler’s expression to profile or line-oriented data (2D source), the x-coordinate is a measure of the distance along the profile, and the y-coordinate is set to zero along the entire profile. Equation 6 is then written in the form of Eq. 7

7 $$ \left( {X - X_{0} } \right)\frac{\partial T}{{\partial X}} + \left( {Z - Z_{0} } \right)\frac{\partial T}{{\partial Z}} = n\left( {B - T} \right) $$

where (x_0_, z_0_) is the position of a 2D magnetic source whose total field T is detected at (x, z). The total field has a regional value of B, and n is a measure of the fall-off rate of the magnetic field. n is directly related to the source slope, is referred to as the structural index, and depends on the geometry of the source^40^.

#### Enhanced horizontal gradient amplitude

Edge enhancement techniques are widely employed to accentuate geologic structures resulting from magnetic field variations^41–43^. A lot of these advanced methodologies for this purpose is extensively documented in existing literature^44,45^.

In this research endeavor, a cutting edge filter based on the derivatives of the horizontal gradient of magnetic data was introduced by^46^ to delineate the lateral boundaries of geologic lineaments. This filter, known as the Edge Enhancement based on Horizontal Gradient Amplitudes (EHGA) operator, can be formally represented as follows8

9 $$P=\frac{\frac{\partial THG}{\partial z}}{\sqrt{{\left(\frac{\partial THG}{\partial x}\right)}^{2}}+{\left(\frac{\partial THG}{\partial y}\right)}^{2}+{\left(\frac{\partial THG}{\partial z}\right)}^{2}}-1$$

where, Ʀ is real part, p is a constant greater or equal to 2, $\left(\frac{\partial THG}{\partial y}\right)+\left(\frac{\partial THG}{\partial y}\right)+\left(\frac{\partial THG}{\partial z}\right)$ directions of the total horizontal gradient (THG) is approximated by:

10 $$THG=\sqrt{{\left(\frac{\partial F}{\partial x}\right)}^{2}+{\left(\frac{\partial F}{\partial y}\right)}^{2}}$$

## Results and discussion

### Remote sensing result

Lineaments are tonal or topographical features on the land that reflect zones of structural weakness^47^. The Central Eastern Desert has been intensively investigated by many workers^21,48^**.** The identification of lineaments has been thoroughly explored by numerous researchers^49–51^. The identification of linear features from remotely sensed data is carried out by applying two main techniques: (1) manual digitizing techniques^47^. (2) Algorithms and software of computers^52^. In this research, Digital Elevation Models (DEM), the first principal component band (PC1) of Landsat-8, and the PALSAR band were utilized to extract lineaments.

#### Lineaments extraction using DEM image

To identify linear topographical characteristics in the Digital Elevation Model (DEM), eight shaded relief images were created by illuminating the image from eight different directions. By using 0 azimuth of solar, we construct the first image of shaded relief. The light directions in the last seven shaded relief images were varied by 45°, 90°, 135°, 180°, 225°, 270°, and 315°.

The input parameters' values determine the number and size of the extracted lineaments. These values are represented by optional digits in the PCI Geomatica software's LINE module. The module's algorithm comprises three steps: edge detection, thresholding, and curve extraction.

**Figure 5:**
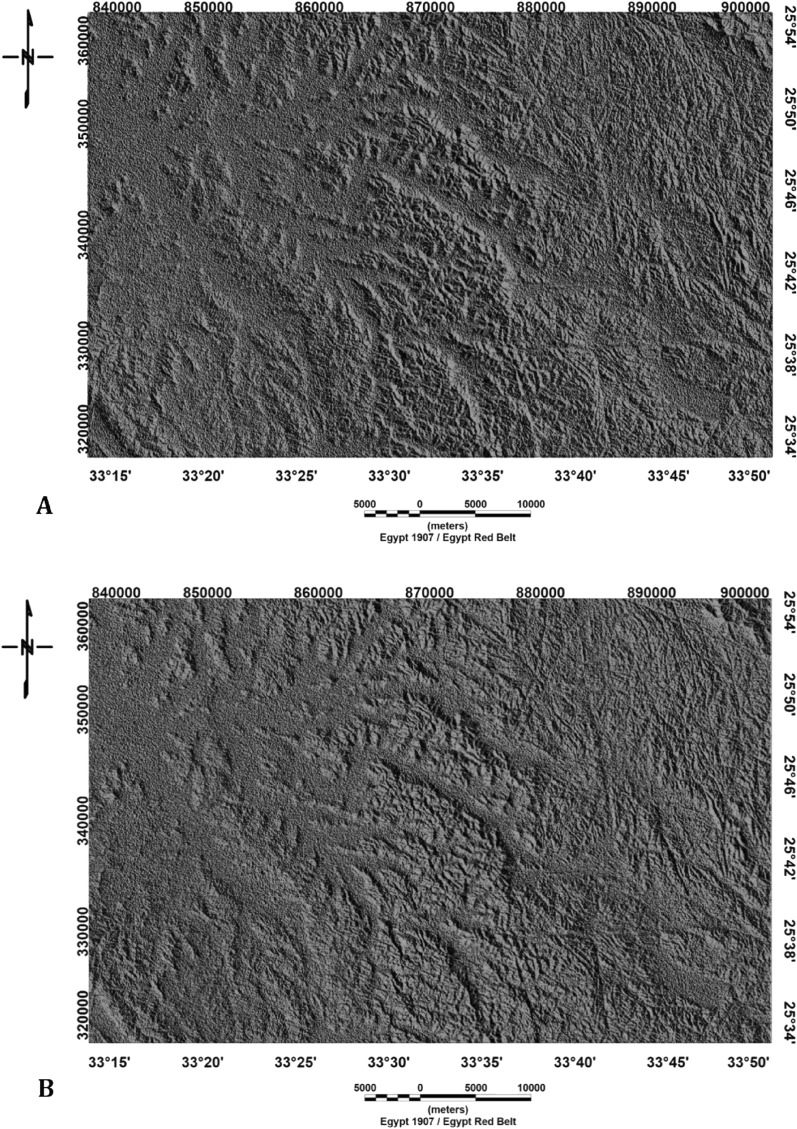
(**A**) Combination of shaded relief images of the Bir El-Qash region, Central Eastern Desert, Egypt, with sun angles of 0°, 45°, 90°, and 135°. (**B**) Shaded relief image generated by integrating four shaded relief images of the Bir El-Qash region in Egypt's Central Eastern Desert with sun angles of 180°, 225°, 270°, and 315°.

**Figure 6:**
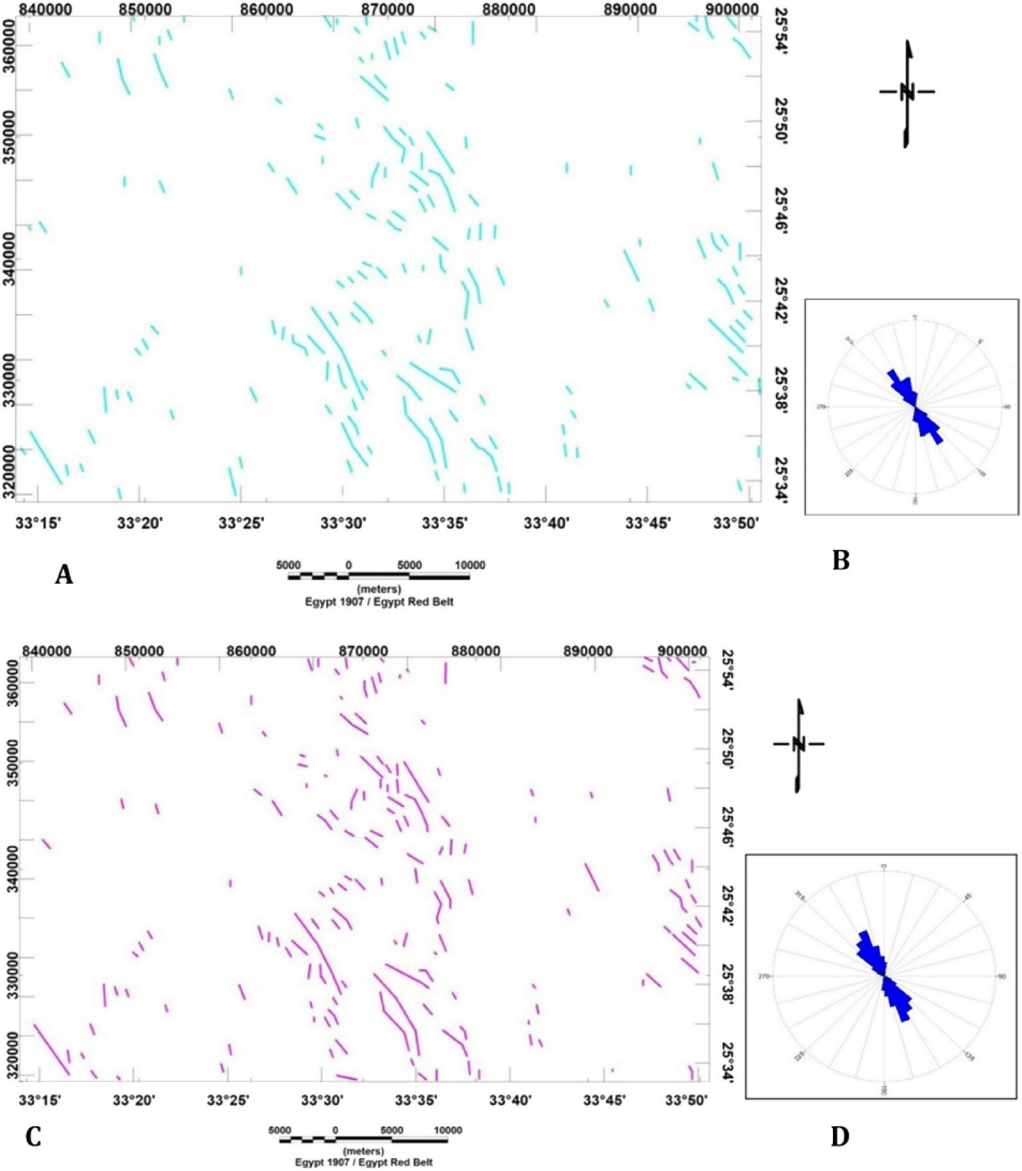
(**A**) Lineament map generated by integrating shaded relief images with sun angles of 0°, 45°, 90°, and 135° and (**B**) Rose diagram of the lineaments of the Bir El-Qash region, Centr of Eastern Desert, Egypt. (**C**) Lineament created by integrating of shaded relief images with sun angles of 180°, 225°, 270°, and 315° and (**D**) Rose diagram of the automated lineaments of Bir El-Qash region, Central of Eastern Desert, Egypt.

However, the LINE module itself is responsible for extracting lineaments from an image and transforming these linear features into vector form, employing six optional parameters. This step involves combining four shaded relief images into a single image^53^ (Fig. 5A, B). This stage entails the creation of four shaded relief images through GIS techniques, achieved by superimposing four shaded relief images to produce a composite image with varying illumination directions (0°, 45°, 90°, and 135°) (Fig. 6A) and another image with multiple lighting directions (180°, 225°, 270°, and 315°) (Fig. 6C). The Geomatica 10.3 software is used to combine the images for automated lineament extraction across the study area. The generated lineaments from both images with different sun angles (0°, 45°, 90°, 135°, 180°, 225°, 270°, and 315°) show an NW–SE trend (Fig. 6B, D).

#### Lineament extraction using OLI data

**Figure 7:**
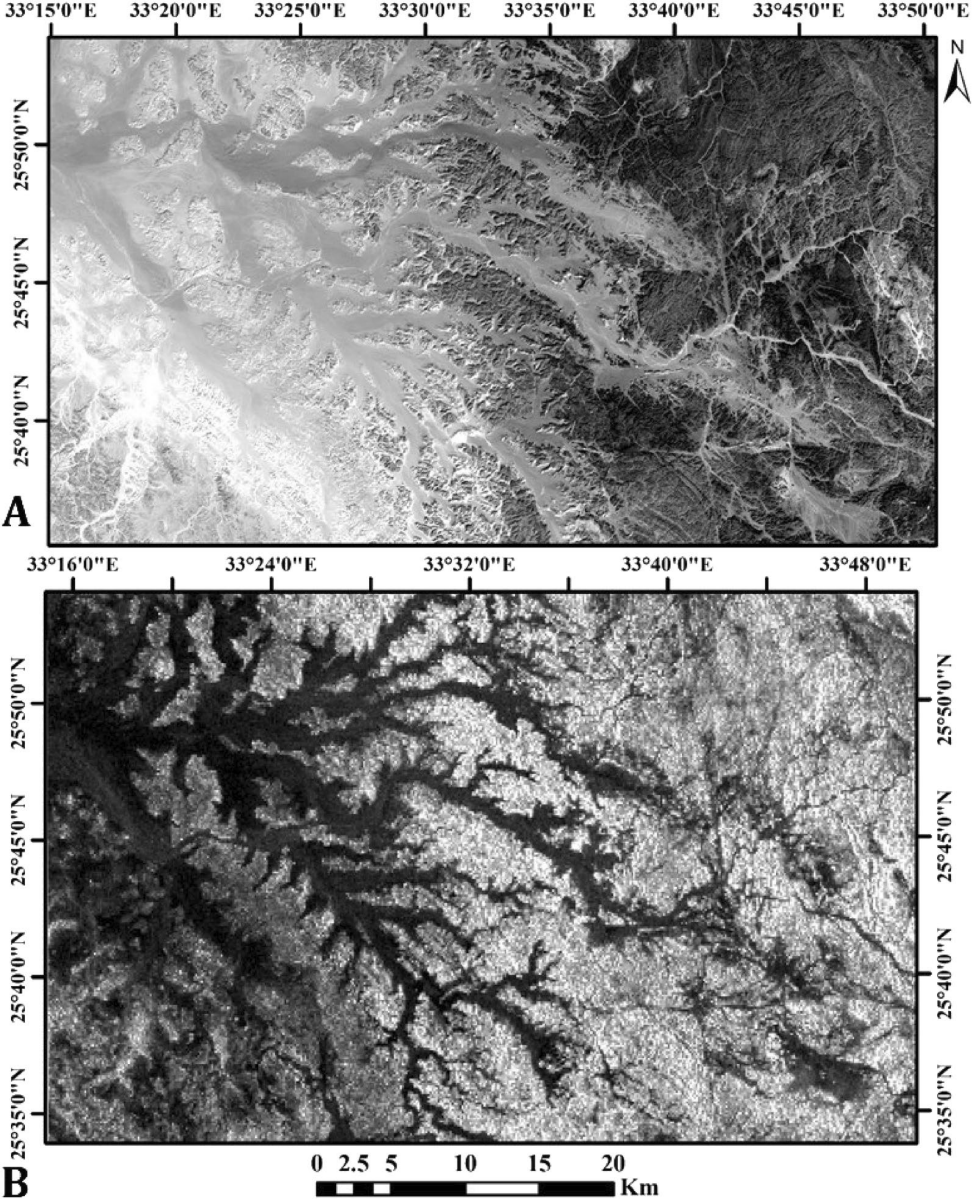
(**A**) Principal Component Analysis (PC1) image of the study area. (**B**) Grayscale image of AlOS/PALSAR SAR data.

**Figure 8:**
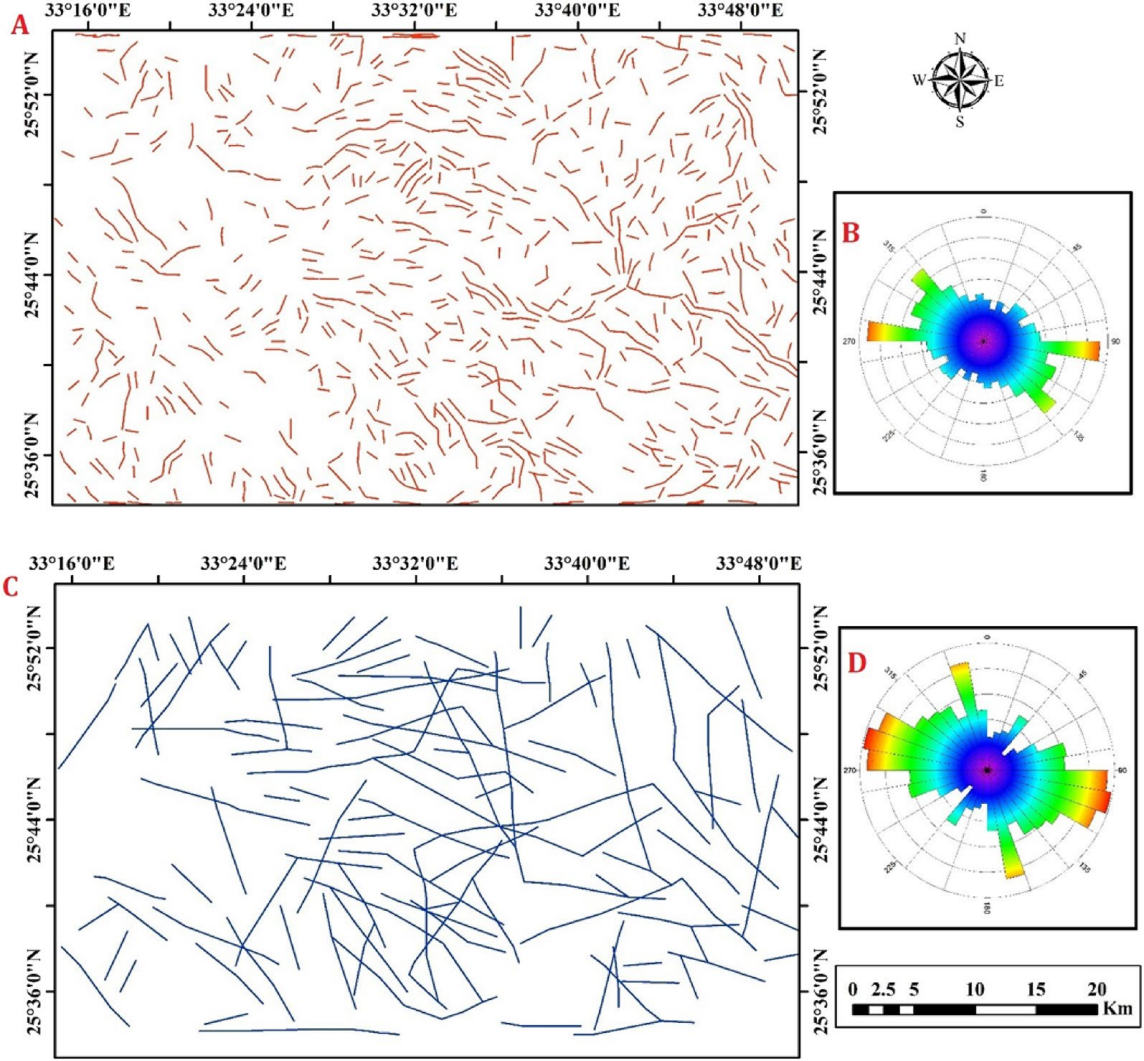
(**A**) Automatic lineament map of PCA1 image of the study area. (**B**) Rose diagram of automatic Lineament map. (**C**) Automatic lineament map of PALSAR image of the study area. (**D**) Rose diagram of automatic Lineament map.

PCA is a widely used multivariate statistical method. The principal component analysis was utilized in this study to separate noise components and reduce the spectral dimensionality of the data, resulting in uncorrelated output bands^54–56^. The PCA technique was applied to OLI data, and the first principal component band (PC1) was chosen for lineament extraction in the study area (Fig. 7A). The results of the principal component analysis (PCA) showed that the first band had a significantly higher variance (96.71%) compared to the other bands (Table 2). Therefore, the PC1 band was selected for the automatic extraction of lineaments in the study area (Fig. 8A).

**Table 2:** The eigenvector values of PCA for OLI band.

Eigenvector	Band 1	Band 2	Band 3	Band 4	Band 5	Band 6	Band 7	Eigenvalues %
PC 1	0.9318	−0.3512	0.08086	−0.0438	0.0002	−0.0004	0.00142	96.71
PC 2	−0.29157	0.8543	−0.4064	−0.1015	0.09809	0.00198	-0.0049	2.27
PC 3	0.19939	−0.2991	0.87965	−0.2182	0.22019	0.02908	−0.0056	0.51
PC 4	−0.025	0.15422	−0.1403	−0.9642	−0.1612	−0.0116	0.01049	0.23
PC 5	−0.0712	−0.16038	−0.1694	−0.0889	−0.75793	0.53086	−0.1187	0.14
PC 6	0.03617	−0.0867	0.07753	−0.04854	−0.5231	0.77822	−0.3218	0.11
PC 7	0.00777	−0.0169	0.0097	0.01441	−0.0748	0.33403	−0.93924	0.03

The lineament analysis of the study area indicates that it has undergone significant tectonic activity, resulting in extensive faulting with varying orientations and magnitudes. Notably, the WNW–ESE and NW–SE trending Najd fault system stands out as a prominent feature (Fig. 8B).

#### Lineament extraction using ALOS/PALSAR image

The ability to outline structural lineaments in radar data has been improved by applying four directional filters (0º, 45°, 90º, and 135°) to the backscatter PALSAR data, which enhances linear features for the extraction process of key structural trends (NE–SW, E–W, and NW–SE, respectively). Additionally, four DEM-derived shaded relief images were extracted from the ALOS/PALSAR DEM which have 12.5 m spatial resolution with four different directions (0º, 45°, 90º, and 135°) to aid in the lineament extraction process with the ALOS/PALSAR data (Fig. 7B). It has been established that when this approach is used together with optical images, it can be trusted as a dependable way to gather information about lineaments and faults^57^. The study area was analyzed using an ALOS/PALSAR image to extract lineaments automatically. Automated lineament extraction was performed using algorithms and computer software e.g.^58–62^. In this research, the PCI Geomatica software was utilized to automatically extract lineaments from the PALSAR image, resulting in vector segments that depict linear features. (Fig. 8C).

The lineament analysis carried out in the research area has provided valuable insights into the tectonic processes that have shaped the geological features of the region. The findings reveal that the area has undergone complex tectonic activity, resulting in extensive faulting with diverse orientations and magnitudes. The identified lineaments include prominent trends such as the WNW–ESE oriented and NW–SE (Red Sea-Gulf of Suez) trend, as well as less conspicuous trends such as the NE-SW oriented Gulf of Aqaba trend. The contradiction arises from the ALOSE/PALSER image, which reveals a WNW–ESE trend. This inconsistency with other study data can be attributed to the unique ability of ALOSE/PALSER to unveil hidden features beneath natural phenomena, employing high-texture lineaments through backscattering radiations. Additionally, it's noteworthy that remote sensing data primarily focuses on surface structures, in contrast to magnetic data, which is concerned with both shallow and deep structures. The interpretation of these lineaments allows for a better understanding of the geological history and evolution of the study area, which may have implications for geological exploration and natural resource management (Fig. 8D).

### Aeromagnetic results

**Figure 9:**
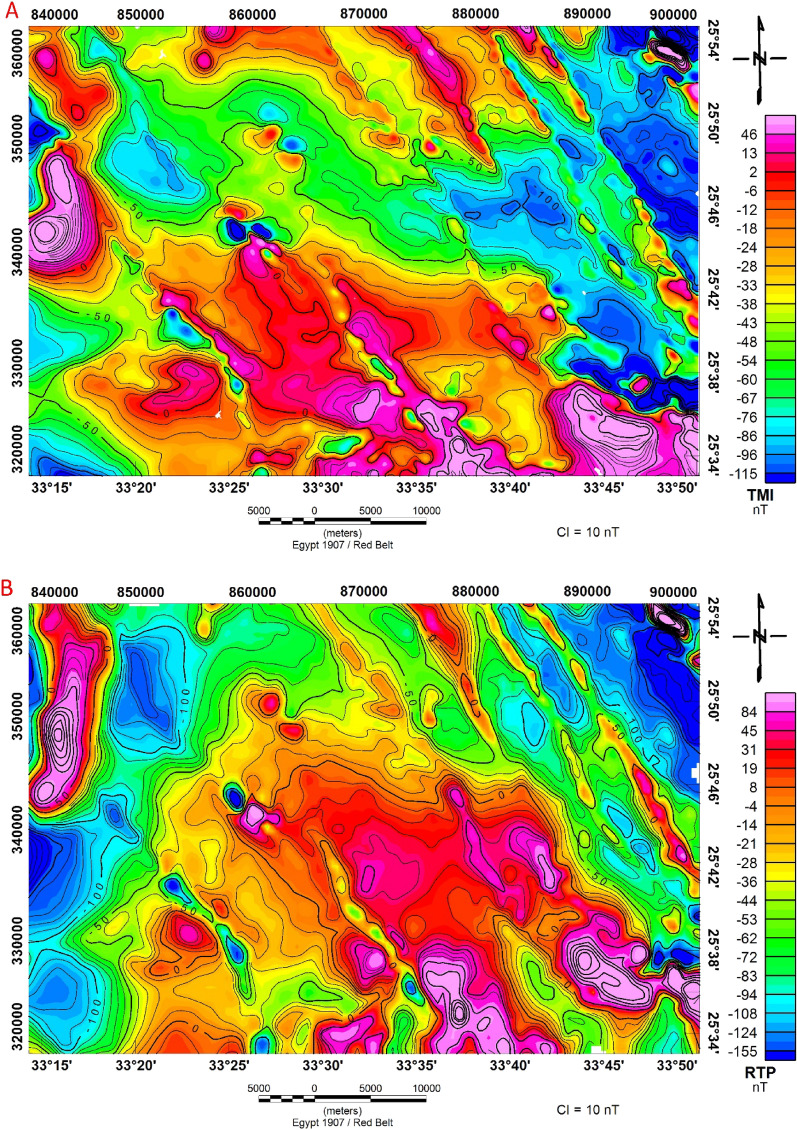
(**A**) Total Magnetic Intensity Map (TMI), and (**B**) Reduced to northern magnetic pole (RTP) map of the of Bir El-Qash area, Central Eastern Desert, Egypt.

The examination of a magnetic map (Fig. 9a) for a specific study area. The map reveals two primary magnetic zones within this area. The first zone, found in both the southern and northern regions of the study area, displays a significant magnetic anomaly with a peak value of 42 nT. This high magnetic anomaly is characterized by high-amplitude variations and is attributed to the presence of high-rise basement outcrops in these areas. The high magnetic values likely result from geological features or materials in the Earth's crust, such as rocks with a strong magnetic susceptibility. The second zone, in contrast, is situated in the central and eastern parts of the study area and is associated with a low magnetic anomaly. This low magnetic anomaly reaches a minimum value of -115 nT. The geological composition of this area is described as sedimentary and acidic rocks. It is also noted that this region trends in an NW–SE direction.

#### Reduced to the north magnetic pole

The total intensity magnetic map (Fig. 9A) was processed by applying 2-D wave number filtration to adapt it to the research area's northern magnetic pole using its inclination and declination. The application of the International Geomagnetic Reference Field (IGRF) technique indicates that the total magnetic intensity is 42,425 gammas in the study area and the angle of the declination and inclination are 39.5 degrees and 2 degrees, respectively. The RTP—magnetic map is created using these parameters to produce the reduced to the north magnetic pole (Fig. 9B). The reduction to the northern magnetic pole has been used to overcome the inclination problems and to locate the magnetic anomalies directly above the causative sources.

Because the inclination effects on the magnetic field at this point have been removed, a general look at this RTP map in comparison to the original total aeromagnetic intensity map shows that the magnetic anomalies appear to have moved northward. The magnetic anomalies on the reduced to the northern magnetic pole map (Fig. 9B) are slightly different from those on the total magnetic intensity (TMI) map (Fig. 9A). In the study area, a magnetic anomaly with a maximum amplitude value of 84 nT has been observed. This anomaly is characterized by positive magnetic anomalies, which are most pronounced in the southern part of the study area. They extend towards the central region and consist of multiple irregular and elongated closures. Furthermore, there's a notable positive magnetic anomaly in the northwest part of the study area, which is elongated and runs in a northwest-southeast direction. Geologically, this area exhibits a diverse range of rock types, including the Quseir Formation, Taref Formation, Hammamat clastics, granites, wadi deposits, metasediments, and ophiolitic serpentinite.

The significant magnetic anomalies might be caused by the existence of subsurface basic intrusions with high magnetic content. The research region exhibits the presence of felsite dykes along Wadi El-Qash in N–S and NE–SW directions that penetrate the basement rocks and molasse deposits and there are felsite sills situated in the northern region of the Zeidun basin. The intrusion of felsite results in the formation of an alteration zone inside the molasse sediments. The Arak basin is affected by NW-trending dykes composed of porphyritic microgranite^16^.

The reduced-to-the-pole magnetic amplitude map demonstrates a notable negative magnetic anomaly with a minimum value of −155 nT. This negative anomaly is primarily located in the western and northeastern sections of the study area. The geological characteristics of these regions include a diverse array of rock types, such as the Taref Formation, Quseir Formation, Duwi Formation, Dakhla Formation, Tarawan Formation, wadi deposits, metavolcanic, and metasediments.

The primary orientation of both positive and negative magnetic anomalies on the map follows two main trends: northwest–southeast (NW–SE) and north–south (N–S). This alignment of magnetic anomalies suggests the presence of structural features within the study area.

#### Radially averaged power spectrum

**Figure 10:**
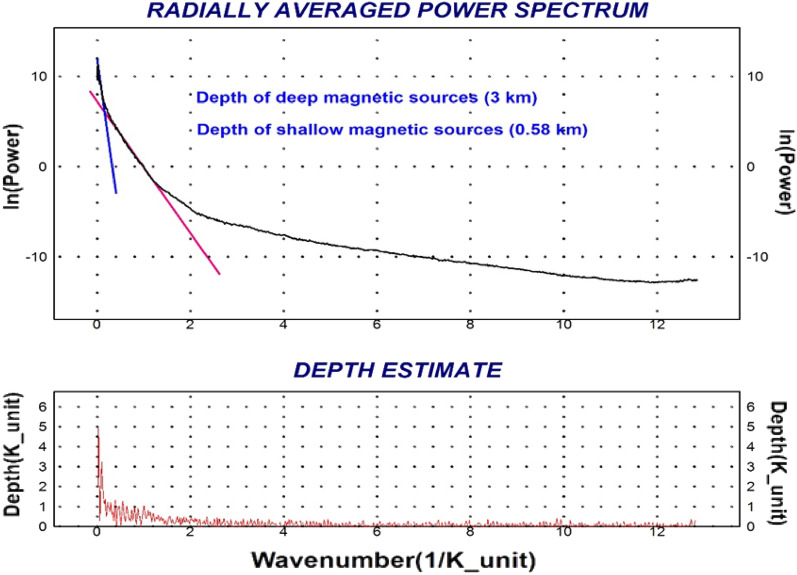
Power spectrum technique shows the depth of magnetic sources of Bir El-Qash area, Central Eastern Desert, Egypt.

The analysis of the two-dimensional power spectrum curve has revealed the presence of two distinct linear segments (Fig. 10). Each of these segments corresponds to specific characteristics of the sources of the observed phenomena.

1. The first segment is associated with deep and/or wide sources. It is characterized by low frequencies, indicating long wavelengths. In other words, the sources contributing to this segment are located at greater depths within the Earth's subsurface.
2. The second segment, on the other hand, is attributed to shallow sources. It exhibits high frequencies, representing short wavelengths. Shallow sources are typically closer to the Earth's surface.

The deep magnetic sources are characterized by wavenumbers ranging from 0.00 to 0.152 cycles per k-unit, and the average depth for regional sources is approximately 3 km. Meanwhile, the shallow sources are associated with wavenumbers ranging from 0.152 to 1.30 cycles per k-unit at the first level of residual, and the average depth for residual sources is approximately 0.58 km.

#### Regional and residual magnetic

**Figure 11:**
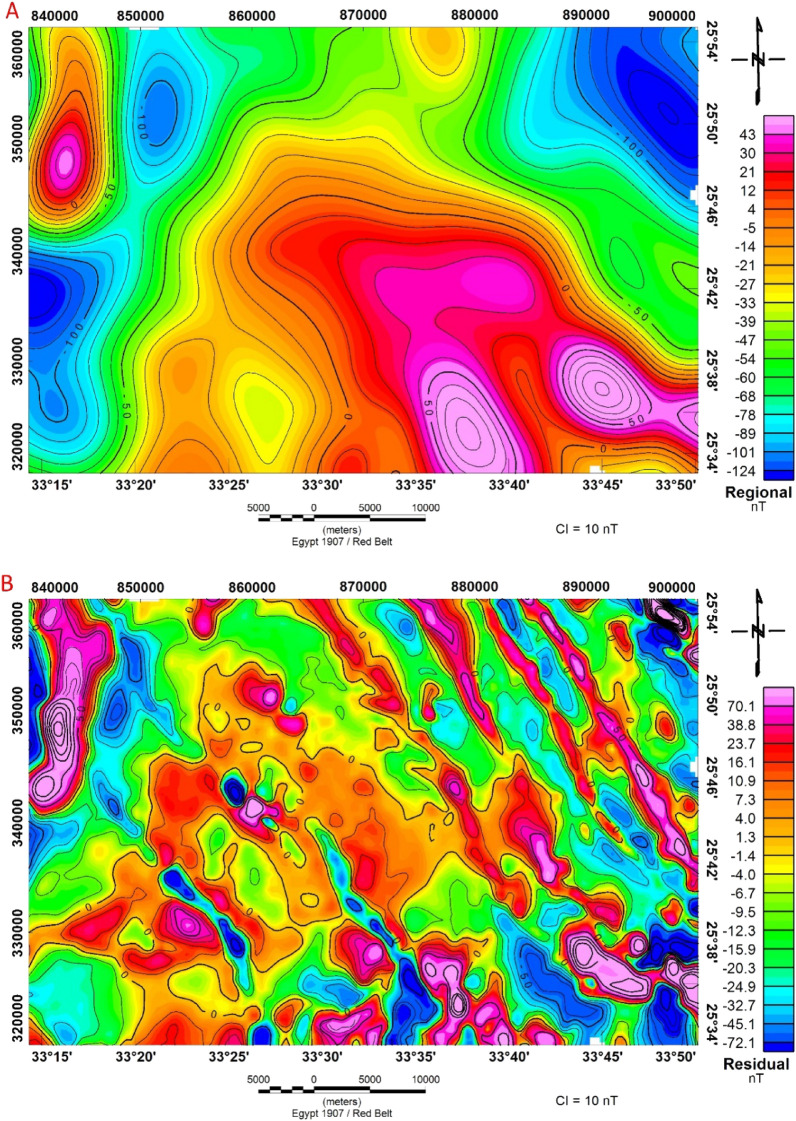
(**A**) Regional anomaly map and, (**B**) Residual anomaly map of airborne magnetic data of Bir El-Qash area, Central Eastern Desert, Egypt.

After removing the residual effects from the RTP map, the regional magnetic component map (Fig. 11A) at the assigned interface is generated. Magnetic anomalies in the region can be separated into two groups. The first group is located in the southern and northwestern parts of the study area. These anomalies have a high range of magnetic values, between 0 and 43 nT. The second group is located in the northeastern and western parts of the study area. These anomalies have a low range of magnetic values, between 0 nT and −124 nT. The main trend of the positive and negative anomalies is NW–SE and N–S.

Geophysicists use residual maps to identify local features that are often obscured by broader features in the field^63^. The residual magnetic anomaly map (Fig. 11B) shows the magnetic field distribution at a shallow depth after removing the regional influence. As a result, the map shows a high frequency of both high and low magnetic anomalies with a limited area of coverage and variations in amplitude and direction. These variations indicate different anomalies, sources, depths, compositions, and structure configurations. The residual magnetic anomaly map (Fig. 11B) shows a pattern of alternating negative and positive magnetic zones, with a general direction of NW–SE, and N–S. This variation in the predominant structural trends (NW–SE and N–S) is attributed to a variety of tectonic processes within the investigated region^12^.

#### First vertical derivative

In data analysis, the first vertical derivative is frequently employed to find edges and geologic limits^64,65^. To improve the shallow sources of geologic features of the data, the vertical derivative is commonly applied to RTP magnetic data. The first or second vertical derivative is often used to emphasize gradients near the edges of shallow magnetic sources. This allows them to be used to find the boundaries of magnetic bodies and identify sources at shallow depths^66^.

**Figure 12:**
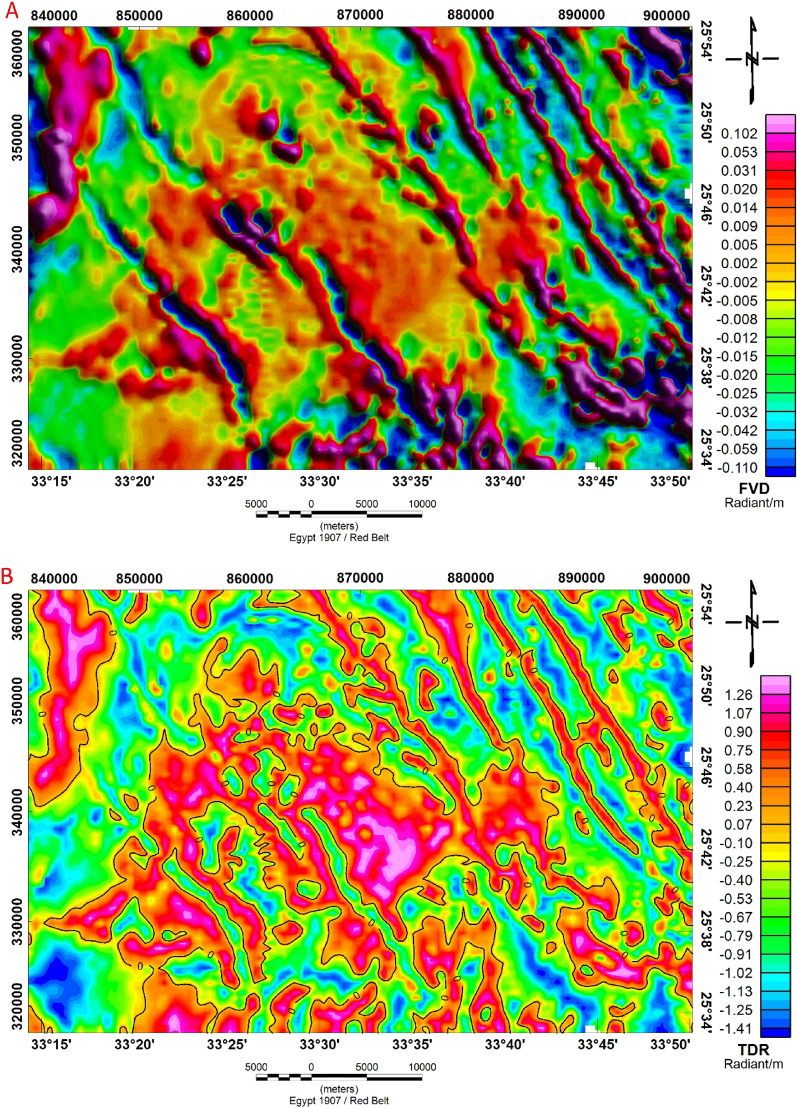
(**A**) First order anomaly map of vertical derivative technique and, (**B**) Tilt Derivative (TDR) technique of Bir El-Qash area, Central Eastern Desert, Egypt.

The analysis of the first vertical derivative technique of airborne magnetic data is shown in Fig. 12A. The NW–SE trending anomaly system is located exactly in the area of an NW–SE striking fault system confined in the major fault lineaments. As a result, the long, narrow, and linear negative magnetic anomalies in particular parts of the area have a strong spatial relationship with fault structures. In other parts of the study area, the direction of elongated and semi-round positive and negative magnetic anomalies aligns significantly with the primary structural trend that affects the research region.

#### Tilt derivative (TDR)

It is feasible to discern numerous unique lineaments in the TDR anomaly map that are very difficult to notice in the total magnetic anomaly field map. The identification of magnetic lineaments is depicted in the TDR anomaly map (Fig. 12B), where the TDR's zero contour is shown in the black line on the tilt derivative map. This map reflects a series of structural lineaments, the NN–SE and NS trends, which represent the main structural trends in the study area and are associated with the Red Sea-Gulf of Suez trend and the East African trend, respectively.

#### (3-D) Euler deconvolution

To create a map that shows the locations and depths of magnetic sources in a two-dimensional grid, 3D Euler deconvolution is used^67^. Any geologic model (faults, magnetic contacts, dykes, sills, etc.) can be used with the Euler Deconvolution method, and it is unaffected by geomagnetic inclination and declination or magnetic remanence^68^. The depth of a magnetic source is crucial for analyzing underlying structures using geological and geophysical methods^55,69,70^. Only when the depth uncertainty of the calculated depth falls beneath a predetermined threshold and the solution's position falls within a predetermined range of the data window's center is the solution deemed to be valid^71^.

Utilizing the RTP magnetic grid of the research region, the depth estimate by 3D Euler deconvolution has been applied^31^. The available RTP anomaly map was subjected to this method using the structural indices (0, and 1.0) and window size equal to 20, as well as a tolerance value of 5.

**Figure 13:**
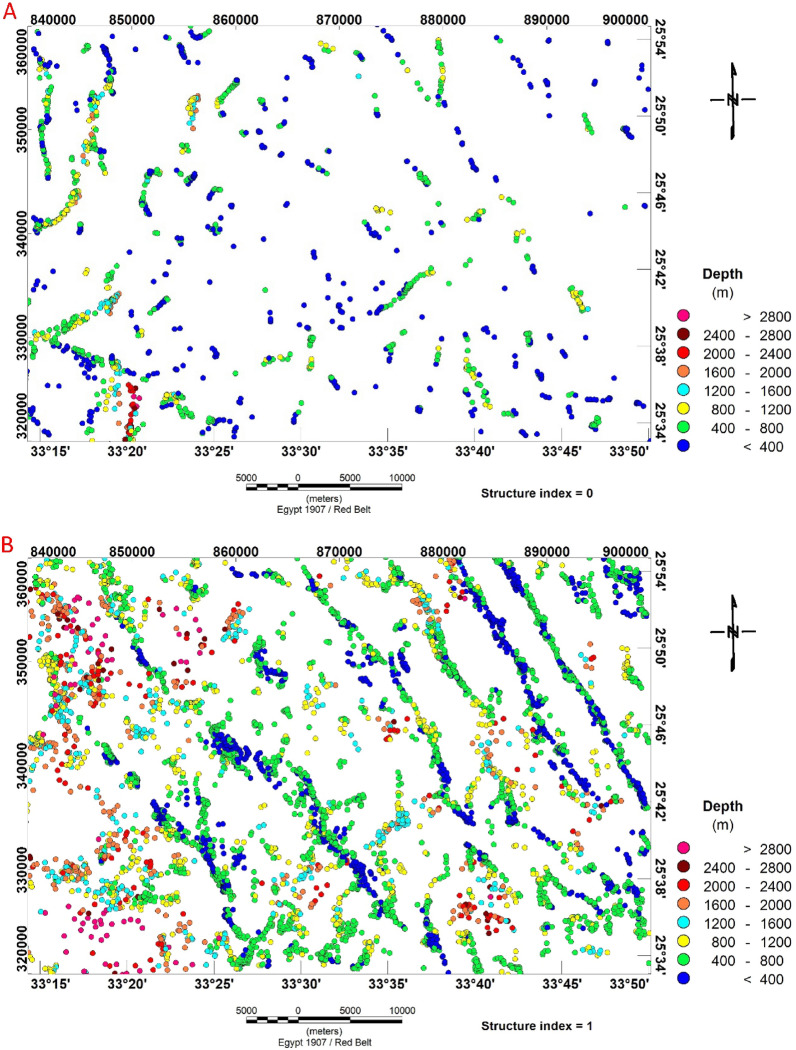
(**A**) Structural index of (0) and (**B**) Structural index of (1) based on the Euler deconvolution technique of Bir El-Qash area, Central Eastern Desert, Egypt.

The simple model of a magnetic field produced by a geological contact or fault structure, concentrated along the borders of the anomalies, is given a structural index of 0. The calculated depth estimates with a structural index of 0 are displayed in (Fig. 13A). According to the Euler deconvolution approach, the basement structural depths in the investigation area range from less than 400 m to more than 2800 m. Additionally, the research shows that the lineament patterns are mainly oriented in the NNW–SSE, NE–SW, and N–S directions, which represents the prevailing structural pattern and tectonic trend of the studied area.

The magnetic dyke or sill solution for structural sources (dykes and sills) is built using RTP magnetic data (Fig. 13B). Using the structural index model (SI = 1), this model provides solutions for the positions and depths of the dykes and sills that intruded the various basement rocks at great depths (Fig. 13B). The depths obtained range from 400 to 2800 m. The analysis of these solutions reveals that the lineament patterns are generally oriented in the NNW–SSE, NW–SE, and N–S directions. These dykes or sills are related to felsite dykes that penetrate the basement rocks and molasse deposits and are situated along Wadi El-Qash and along the northern region of the Zeidun basin^16^.

#### Enhanced horizontal gradient amplitude

The EHGA technique can generate well defined edges for magnetic sources. The peaks of the EHGA are located directly over the source borders, and this filter generates more reliable responses. The EHGA filter is applied to the aeromagnetic data and clearly outlines and refines the lineaments of the studies region.

**Figure 14:**
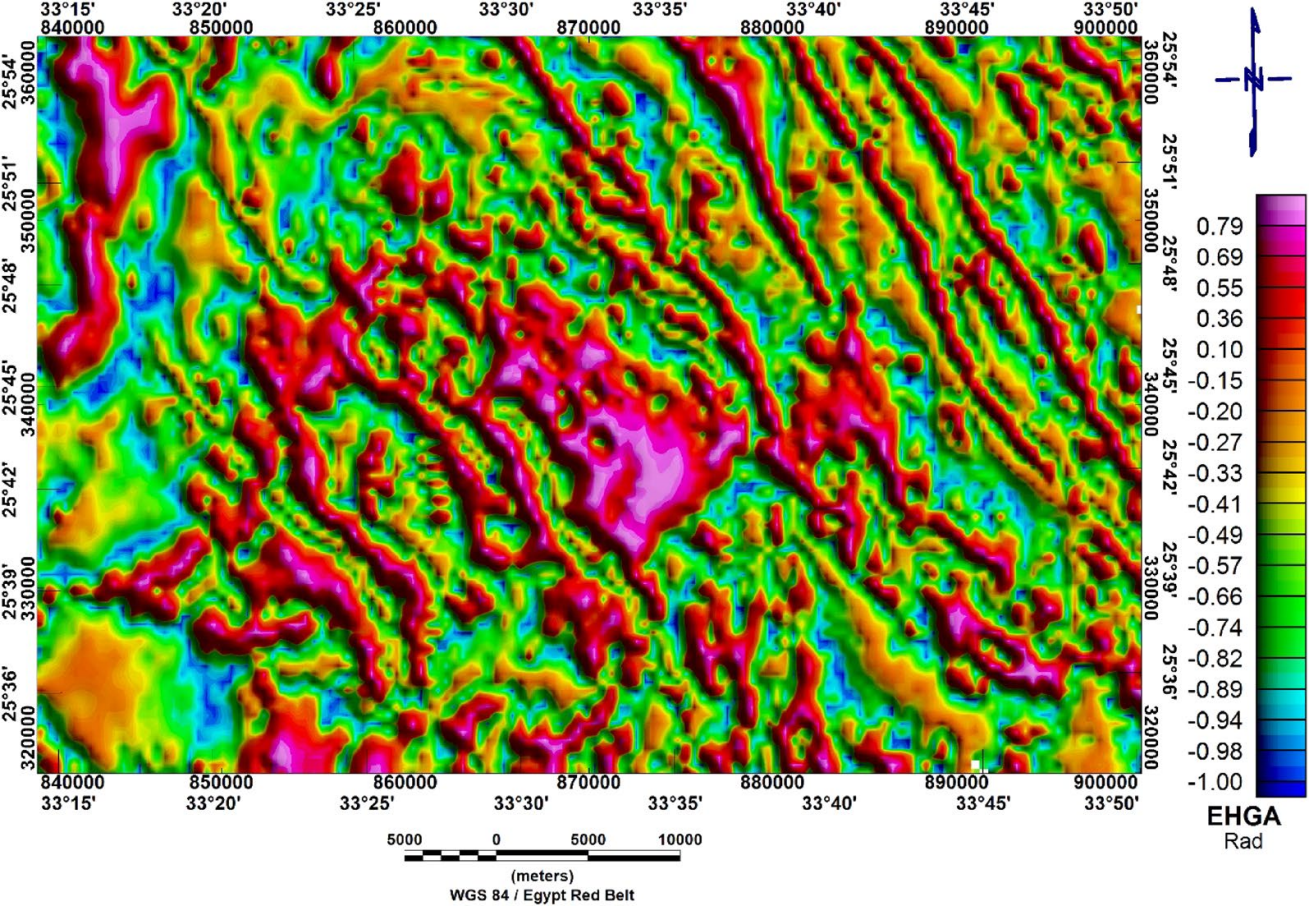
Enhanced horizontal gradient amplitude of Bir El-Qash area, Central Eastern Desert, Egypt.

The Enhanced horizontal gradient amplitude map (Fig. 14) shows alternating between positive (high) and negative (low) values. The positive values reach up to 0.79 rad, while the negative values reach to -1rad. The areas with high amplitude suggest stronger magnetic gradients. This could be indicative of geological feature such as faults, changes in rock type or mineral deposits that exhibit significant contrasts in magnetic propereties. The analysis and interprtation of EHGA shows that the study area is highly deformed and the main structural trending the NW–SE, NNW–SSE and N–S directions.

#### Structural interpretation

Finding linkages between the data and the region's subterranean structure is a key aspect of interpreting potential field data in geology. In several areas of geology and geophysics, structural difficulties are frequently found through analyzing structural patterns. A significant relationship between the pattern, strength, and direction of magnetic anomaly trends was discovered^66^. This is due to the fact that the presence and strength of magnetic contrasts in the rocks have a significant impact on the visibility of faults on magnetic maps.

**Figure 15:**
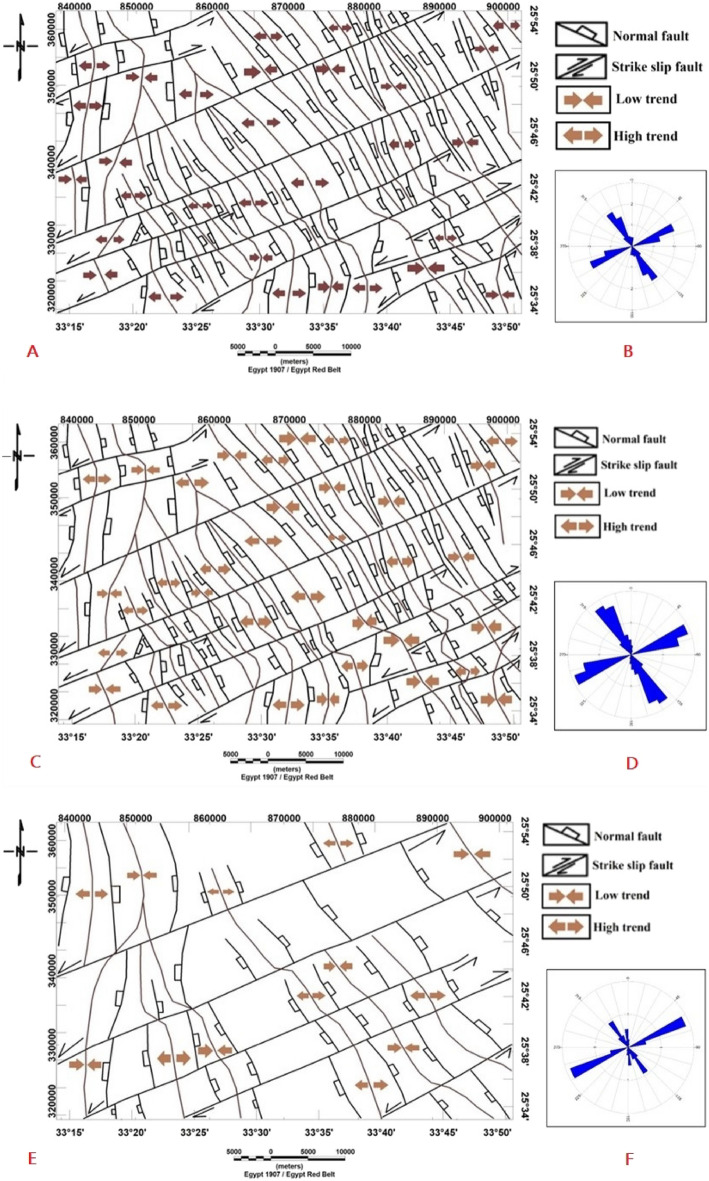
(**A**) Structural elements resulting from tilt derivative map, (**B**): rose diagram of structural elements of tilt derivative map, (**C**) shallow structural elements, (**D**) rose diagram of shallow structural elements and, (**E**) deep-seated structural elements and, (**F**) rose diagram of deep-seated structural elements of the study area.

The region under investigation is primarily composed of igneous and sedimentary rocks that have been impacted by various types, trends, and positions of regional and local geological structures. The interpreted magnetic structure maps of the study area were constructed using surface geologic information, the interpretation of magnetic maps, and the research results of the structural trend analysis. Several magnetic methods were used to extract the subsurface structures in the study area, such as tilt derivative structural elements (Fig. 15A), shallow-seated structural elements (Fig. 15C), and deep-seated structural elements (Fig. 15E).

The analysis of these diagrams reveals three primary structural trends of varying degrees of strength and duration. According to magnetic data, these three trends (NW, NE, and N-S) are the most significant structural trends affecting the area under study. As illustrated below, each of the major trend categories is briefly discussed in descending order. The trend analysis of the interpreted magnetic structural maps is used to explore their relationship in the present area and obtain an idea about the main features of its structural pattern. Rose diagrams (Fig. 15B, D, F) were built to varying degrees from available data to aid in the understanding of structural trends affecting the study area. These diagrams reveal two major structural trends: the Red Sea-Gulf of Suez trend (NW–SE) and the Syrian arc trend (NE-SW). Meanwhile, the Meridional or East African (N–S) trend appears as a minor trend. Each of the major and minor trend categories is described briefly, as shown below.

#### Syrian arc trend (NE–SW)

The magnetic anomaly maps reveal the NE-SW trend as the dominant trend. The analysis of the rose diagrams of the regional, residual, and tilt derivative structure maps (Fig. 15) indicates that this trend has similar azimuth and intensity significance and is considered a strong trend on the deep- and shallow structural elements maps (Fig. 15). This trend is known as the Aualitic trend and is linked to rift tectonics in the Gulf of Aqaba^73^. He also noted that it is less prominent near the stable shelf and characterizes several of Egypt's fold systems in the middle (Syrian arc system). The Jordan-Dead Sea-Aqaba rift has been active as a strike-slip fault since the Oligocene period and has evolved into a graben since the late Pliocene^74^.

#### Red sea-gulf of Suez trend (NW–SE)

It is the most prevalent one in the area at all depths presented by the geologic and processed maps. In addition to its surfacial significance, the NW–SE trend is a significant deep-seated tectonic trend. This trend appears to be a dominant trend of both deep and shallow structures in the Nile Valley, Sinai, and the Gulf of Suez^75^. He further said that the large extent of this direction, as well as its occurrence in different depth zones of the crust, give the idea that it is associated with an old tectonic movement that has lately been reactivated, most likely with the movement created in the Aqaba direction. The peaks of the NW trend were identified as a major trend in the shallow-seated structural elements map (Fig. 15C); however, this trend was identified as a moderate tilt derivative structure map (Fig. 15A) and a deep-seated structural elements map (Fig. 15E).

#### East African trend (N–S)

This trend encompasses all trends that run from N 11.25°E to N 11.25° W and is known as the "East-African trend^73^. The N–S trend is specifically highlighted on the rose diagram of deep-seated structural elements (Fig. 15F) indicating its significance in the deeper parts of the study area. However, it's worth noting that this north–south trend is not prominently observed in the shallow depths (Fig. 15B, D) It is a prominent Precambrian direction as well as a tensional late Tertiary direction^75^ and is considered the oldest fracture system in the Eastern Desert^63^. The Nubia trend, which is found in the basement rocks of the Eastern Desert, Sinai, and inliers in southern Egypt, is thought to be the northern extension of the Mozambique belt^76^. The (N–S) trend is also visible as a main trend on the surface structure map.

## Conclusions

This paper deals with the evaluation of the surface and subsurface structure of Bir El-Qash Area, Central Eastern Desert, Egypt, from DEM and aeromagnetic data. A digital elevation model (DEM), Landsat-8 Operational Land Imager (OLI) data, and Phased Array Type L-Band Synthetic Aperture Radar (PALSAR) images are the three types of remote sensing data that are used to find surface lineaments. Meanwhile, the subsurface lineaments were detected using aeromagnetic data. The results from the analysis of remote sensing data and field geology revealed four main trends, namely NW–SE, WNW–ESE, NE–SE, and N–S. The results demonstrate that the ALOS/PALSAR SAR image is the most effective data source for extracting surface structures. The striking differences in the results of the previous structural map of the study area arise WNW–ESE trend revealed only in the ALOSE/PALSER image. The divergence from prior research outcomes can be attributed to the unique capacity of ALOSE/PALSER to expose hidden features within natural phenomena, utilizing high-texture lineaments through backscattering radiations. Additionally, it is noteworthy to highlight that remote sensing data primarily focuses on surface formations, in contrast to magnetic data, which encompasses both shallow and deep formations.

Initially, the total intensity aeromagnetic map of the study area was transformed to the northern magnetic pole (RTP) to identify the magnetic anomalies' location above their underlying sources. Regional-residual separation techniques were applied to the RTP magnetic map to differentiate between shallow anomalies (structures) and deeper ones. This separation was performed in the frequency domain using the Fast Fourier transformation, which is implemented by analyzing the energy spectrum of the magnetic maps. Through power spectral analysis, the RTP anomaly map of the study area was separated into residual and regional magnetic anomalies.

Spectral analysis, and Euler deconvolution are all automatic source depth estimation techniques that may be used to map the basement depth. The analysis of spectral data from the research area's aeromagnetic data showed two major magnetic anomaly sources depths, deep magnetic sources, and shallow magnetic sources. The deep source anomalies are located at a depth of 3km, which corresponds to the magnetic basement depth. At a depth of 0.58km. Based on the interpretation of the EHGA, residual, regional, and TDR techniques, the study area reveals pronounced deformation, with predominant structural orientations primarily observed along the NW–SE, NNW–SSE, and N–S.

In summary, the study has identified three major structural trends within the investigated area through the examination magnetic and remote sensing data. The geological framework of the region under consideration is marked by prominent trends, namely, the Red Sea-Gulf of Suez trend, the Syrian Arc trend, and the WNW–ESE trend. These identified trends are considered to be the most dominant and influential tectonic features in the study area. They have played a crucial role in shaping the geological characteristics of the region, including mineral distributions.

In contrast, the Meridional or East African (N–S) trend appears to be of minor significance compared to the Red Sea-Gulf of Suez and Syrian Arc trends. While it may still have an impact on the area's geological features, it is not as prominent or influential as the other two major trends.

## Data Availability

The data presented in this study are available on request from the corresponding author.
